# Molecular investigation on *Listeria monocytogenes* and *Salmonella enteritidis* in table eggs and diarrheic patients

**DOI:** 10.1038/s41598-026-66486-1

**Published:** 2026-08-20

**Authors:** Asmaa A. Rayan, Reem M. Alsaadawy, Marwa G. Abd El Kader

**Affiliations:** 1 Department of Microbiology and Immunology, Faculty of Veterinary Medicine, Assiut University, Assiut, 71526 Egypt; 2 Department of Zoonoses, Faculty of Veterinary Medicine, Assiut University, Assiut, 71526 Egypt; 3 Department of Food Hygiene, Safety and Technology, Faculty of Veterinary Medicine, Assiut University, Assiut, 71526 Egypt

**Keywords:** Eggs, *L. monocytogenes*, *S. enteritidis*, PCR, Stool, Diseases, Microbiology

## Abstract

*Listeria monocytogenes* (*L. monocytogenes*) is a widely distributed zoonotic foodborne pathogen capable of surviving under harsh environmental conditions, including low pH and high salt concentrations. Eggs and egg products represent one of the most common sources of human salmonellosis, primarily caused by *Salmonella enterica* serovar *Enteritidis* (*S. enteritidis*). Accordingly, the present study was conducted to perform a molecular investigation of *L. monocytogenes* and *S. enteritidis* in table eggs and stool samples obtained from diarrheic patients. A total of 58 pooled samples of fresh hen’s eggs, along with 34 stool samples, were collected and subjected to DNA extraction followed by polymerase chain reaction (PCR) analysis. The results revealed the presence of *L. monocytogenes* and *S. enteritidis* DNA in 55.2% and 50% of the examined egg samples, respectively. A higher prevalence of *L. monocytogenes* was detected on eggshells compared to egg contents. Variations in infection rates were observed based on gender and age; *L. monocytogenes* infections were more frequently identified in males and younger individuals, whereas *S. enteritidis* infections were more common among females and older children. Gastrointestinal symptoms were associated with both infections, with abdominal pain being particularly prevalent in patients infected with *S. enteritidis*. In conclusion, the findings of this study highlight the potential zoonotic risk posed to consumers and underscore the urgent need for implementing effective control programs in laying hen farms.

**Supplementary Information:**

The online version contains supplementary material available at 10.1038/s41598-026-66486-1.

## Introduction

Microbial contamination of table eggs has significant implications for the poultry industry, while egg-associated illnesses represent a serious public health concern worldwide. Eggs can serve as important vehicles for the transmission of foodborne pathogens^1^. Table eggs, including raw and ready-to-eat products stored under refrigeration, have been reported to harbor *Listeria monocytogenes* (*L. monocytogenes*), a zoonotic foodborne bacterium responsible for listeriosis in humans^2–4^. Contamination of foods with *L. monocytogenes* has been associated with numerous sporadic cases and large-scale outbreaks globally. Infection with this pathogen can result in a wide range of clinical manifestations, including central nervous system disorders, septicemia, endocarditis, localized infections, and gastroenteritis. In vulnerable populations, particularly pregnant women, it may also lead to severe outcomes such as stillbirths and miscarriages^5^.

Invasive listeriosis represents the more severe form of the disease and primarily affects high-risk groups, including pregnant women, the elderly, immunocompromised individuals, and neonates^6^. The severity of infection is influenced by both the virulence of the infecting strain and the immune status of the host. Although relatively uncommon, listeriosis is associated with a high case-fatality rate, with mortality estimates ranging from 20% to 30% annually^7–9^.

The epidemiology of *Salmonella enterica* serovar *Enteritidis (S. enteritidis)* highlights its strong adaptation to table eggs as a major biological reservoir. Since the mid-1980s, S. enteritidis has been responsible for the majority of zoonotic foodborne salmonellosis outbreaks worldwide. In the United States, 298 (80%) of the 371 outbreaks with identified sources reported between 1985 and 1999 were attributed to eggs^10,11^. Similarly, in the European Union, 165,023 confirmed cases of human salmonellosis were reported in 2006 through the European Surveillance System (TESSy)^12^. Among these, *S. enteritidis* accounted for approximately 62.5% of cases, whereas *Salmonella Typhimurium* was responsible for about 12.9%. Other serotypes collectively contributed to a smaller proportion of infections, with Infantis, Virchow, Newport, Hadar, Stanley, Derby, Agona, and Kentucky among the top reported causes. Notably, eggs and egg products were the most frequently implicated food vehicles in salmonellosis outbreaks^13^. These findings emphasize the strong epidemiological link between *S. enteritidis* infections in humans and the consumption of contaminated eggs and egg-derived products.

Protective measures, including the establishment of effective surveillance and detection systems for food safety, are essential to ensure the safety of both raw and processed animal-derived foods, such as table eggs, for human consumption. Among commonly consumed foods, whether at home, in restaurants, or from convenience outlets, table eggs are distinctive in that they are often served in their natural form without the addition of artificial additives. Under proper flock management and in the absence of vertical transmission, most freshly laid eggs are internally sterile. This is largely attributed to the presence of natural protective barriers, including the cuticle, shell membranes, and the inherent antimicrobial properties of eggs^14^.

Conventional culture-based methods for detecting foodborne microorganisms are time-consuming, as they involve multiple steps, including enrichment, selective plating, and incubation. In contrast, polymerase chain reaction (PCR) has become one of the most widely used diagnostic tools due to its rapidity, high sensitivity, and specificity, enabling the prompt detection and identification of specific pathogens from diverse sources^14–16^. Accordingly, the present study was conducted to determine the prevalence of the foodborne pathogens *L. monocytogenes* and *S. enteritidis* in table eggs and stool samples from diarrheic patients in Assiut City, Egypt. In addition, the study aimed to evaluate associated risk factors, including age and sex, using molecular detection methods.

## Materials and methods

### Ethical approval

The experimental protocol of this study was performed in accordance with the relevant guidelines and regulations of the Scientific Research Committee and the Ethics Board of the Faculty of Medicine, Assiut University, Assiut, Egypt, which approved all procedures of the study (Ethical Approval No. 40–2025-201495). Written informed consent was obtained from all human participants.

### Sample collection and preparation

#### Collection of egg samples

Fifty-eight fresh hen`s egg samples (pooled samples), including farm eggs and Baladi (farmer´s house) eggs, were commercially purchased directly from local supermarkets for molecular detection of *L. monocytogenes* and *S. enteritidis* on egg shells and contents (29 pooled samples for each type, each pooled sample was represented by three eggs)^17^. Cracked, improperly stored, contaminated, unlabeled, or inadequately collected samples that could compromise laboratory results were excluded from the study. Egg samples were obtained using a systematic random sampling approach to ensure representative selection and minimize selection bias. Sampling was conducted across multiple supermarkets located in different areas of Assiut city, and visits were performed on different days and times throughout the study period (June to November 2025) to account for variability in supply sources. These samples were transferred in plastic bags in a tank to the laboratory. Whole eggs were stored in a refrigerator at 4^◦^C until DNA extraction.

#### Preparation of egg samples^17^

The three eggs of each pooled egg sample were washed in 15 mL of sterile saline in a sterile beaker of 1000 mL capacity, rubbed with a sterile swab, and taken as an eggshell sample in a sterile Falcon tube of 15 mL, from which 200 µl was taken in a sterile Eppendorf tube for DNA extraction. The three eggs were sprayed with 70% alcohol and exposed to flame on their broad ends, and aseptically broken; their contents were taken into a sterile beaker and thoroughly homogenized and mixed by a sterile fork. 200 µl was taken in a sterile Eppendorf tube as an egg content sample. The eggshell and egg content samples were stored at −20 °C until DNA extraction. This part of the work was done in the Department of Food Hygiene, Safety and Technology, Faculty of Veterinary Medicine, Assiut University, Assiut, Egypt.

#### Collection of stool samples

Thirty-four stool samples were collected from diarrheic human patients aged 5 months to 16 years, and included 20 males and 14 females from different hospitals and medical laboratories in Assiut city, Egypt, from June to November 2025. Following clinical assessment, the study objectives were explained to the patients’ guardians, and written informed consent was obtained prior to sample collection. A structured epidemiological questionnaire was then administered to collect demographic data (age and sex) and relevant clinical history, including gastrointestinal symptoms and recent illness. Samples from patients without diarrhea and samples with insufficient quantity or those not transported and stored according to the study protocol were excluded.

The stool samples were collected in sterile cups, transferred to the laboratory, and preserved at −20^◦^C until molecular examination for the presence of *L. monocytogenes* and *S. enteritidis* infection.

### Molecular detection

#### DNA extraction

From egg shell, content, and stool samples, DNA extraction was performed according to the manufacturer’s instructions for the ABT Genomic DNA Mini Extraction Kit (Cat. No. EX01). The whole DNA of the samples was extracted and stored at −20 °C.

#### PCR amplification and primer design

PCR was used to amplify the *L. monocytogenes* genome’s hemolysin (*hlyA*) gene with a length of 234 bp PCR products and *S. enteritidis* genome’s *Salmonella enteritidis* phage type 4 strain with length of 250 bp PCR products were produced. The total volume of the PCR reaction was 20 µl, the reaction contained the following: 10 µl 2X ABT red master mix (Applied Biotechnology, Egypt) (Cat. No. AMP 01), 1 µl of each forward and reverse primers (10 pmol), and 8 µl template DNA. The conditions for primers during PCR for *L. monocytogenes* detection were started with initial denaturation at 95˚C for 10 min, followed by 45 cycles including the denaturation phase at 95˚C for 20 s, annealing phase for 30 s at 60 °C, and extension phase at 72˚C for 30 s. At the end, the final extension was carried out at 72˚C for 7 min., while the conditions for primers during PCR for *S. enteritidis* detection were started with initial denaturation at 94˚C for 5 min, followed by 45 cycles including the denaturation phase at 94˚C for 30 s, annealing phase for 30 s at 58 °C, extension phase at 72˚C for 30 s. At the end, the final extension was carried at 72˚C for 10 min. The PCR amplification was performed in a thermal cycler (Kyratec-Thermal Cycler, Adelab Scientific, Australia). Primers for detecting DNAs of the *L. monocytogenes* and *S. enteritidis* directly in samples were developed as shown in Table 1.

**Table 1:** The primers used in PCR reactions.

Bacteria spp.	PCR target and gene	Primer name (internal EURL no.)	T_m_ (°c)	Sequence (5′ to 3′)	Size (bp)	Reference
*L. monocytogenes*	Hemolysin (*hlyA*) gene	LM1F	59°c	5′CGGAGGTTCCGCAAAAGATG 3′	234 bp	^43,44^
		LM2 R		5′CCTCCAGAGTGATCGATGTT 3′		
*S. enteritidis*	*Salmonella enteritidis* phage type 4 strain	S1	58°c	5′ GCCGTACACGAGCTTATAGA 3′	250 bp	^45,46^
		S4		5′ ACCTACAGGGGCACAATAAC 3′		

#### Gel electrophoresis

For visualization, 8 µl of amplified PCR products were subjected to 40 min of gel electrophoresis at 150 V and 116 mA in a 2% agarose molecular grade gel, from GENETIX biotech ASIA PVT.LTD. (Cat.:PG-40005). It was prepared according to^18^ and then mixed with ethidium bromide solution 0.5 µg/mL (2 µl/100 ml gel) (applied Biotechnology, Cat.No.EL01) for electrophoresis using Electrophoresis Equipment (Cleaver Scientific & multiSUB Midi, Midi Horizontal Electrophoresis System, UK). The gel was documented and visualized on a UV transilluminator (Gel Imager - Azure 200, USA); to view the amplicons after their size was determined using size marker DNA of 100 bp (BERUS 100 bp, WF-10407002). The part of molecular detection was made in the Department of Microbiology and Immunology, Faculty of Veterinary Medicine, Assiut University, Assiut, Egypt.

### Statistical analysis

All statistical analyses were performed using the Statistical Package for the Social Sciences (SPSS) software (Version 16.0; SPSS Inc., Chicago, IL, USA). Descriptive statistics were used to summarize the prevalence of *L. monocytogenes* and *S. enteritidis* in egg samples (farm and Baladi) and stool samples from diarrheic patients. Associations between categorical variables (e.g., egg type, sex, age group, and clinical symptoms) and the detection of the target pathogens were assessed using the Chi-square (χ²) test. A *p* < 0.05 was considered statistically significant.

## Results

### Molecular detection of L. monocytogenes and S. enteritidis in egg samples

#### Prevalence of L. monocytogenes and S. enteritidis in egg samples

As shown in Table 2, positive *L. monocytogenes and S. enteritidis* DNA was detected in 55.2% and 50% egg samples, respectively, with no significant difference.

**Table 2:** Prevalence of *L. monocytogenes* and *S. enteritidis* infections in egg samples.

Bacteria spp.	No. of examined samples	Positive (%)	X_2_	*p* < 0.05
*L. monocytogenes*	58	32 (55.2%)	^0.311^	^0.577^
*S. enteritidis*	58	29 (50%)		

#### Prevalence of L. monocytogenes and S. enteritidis in egg types and parts

**Fig. 1:**
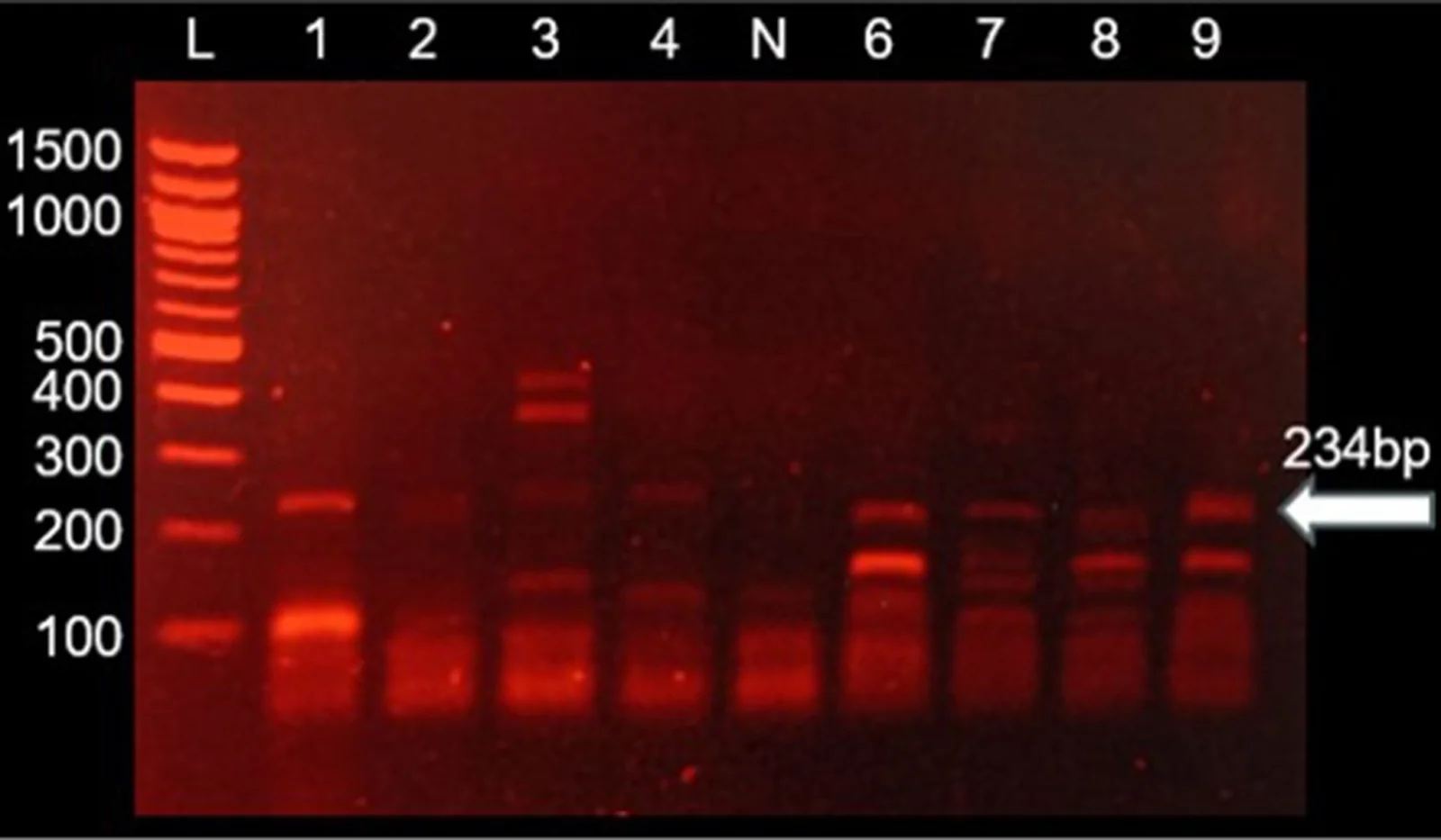
Amplification of *L. monocytogenes* genome’s hemolysin (*hlyA*) gene with a length of 234 bp PCR products. Lane L: DNA ladder (100 bp), lane 1: positive *L. monocytogenes* stool sample, lane 2: positive *L. monocytogenes* farm egg content sample, lane 3: positive *L. monocytogenes* farm egg shell sample, lane 4: positive *L. monocytogenes* Baladi egg content sample, lane N: negative control, and lanes 6–9: positive *L. monocytogenes* Baladi egg shell samples.

**Fig. 2:**
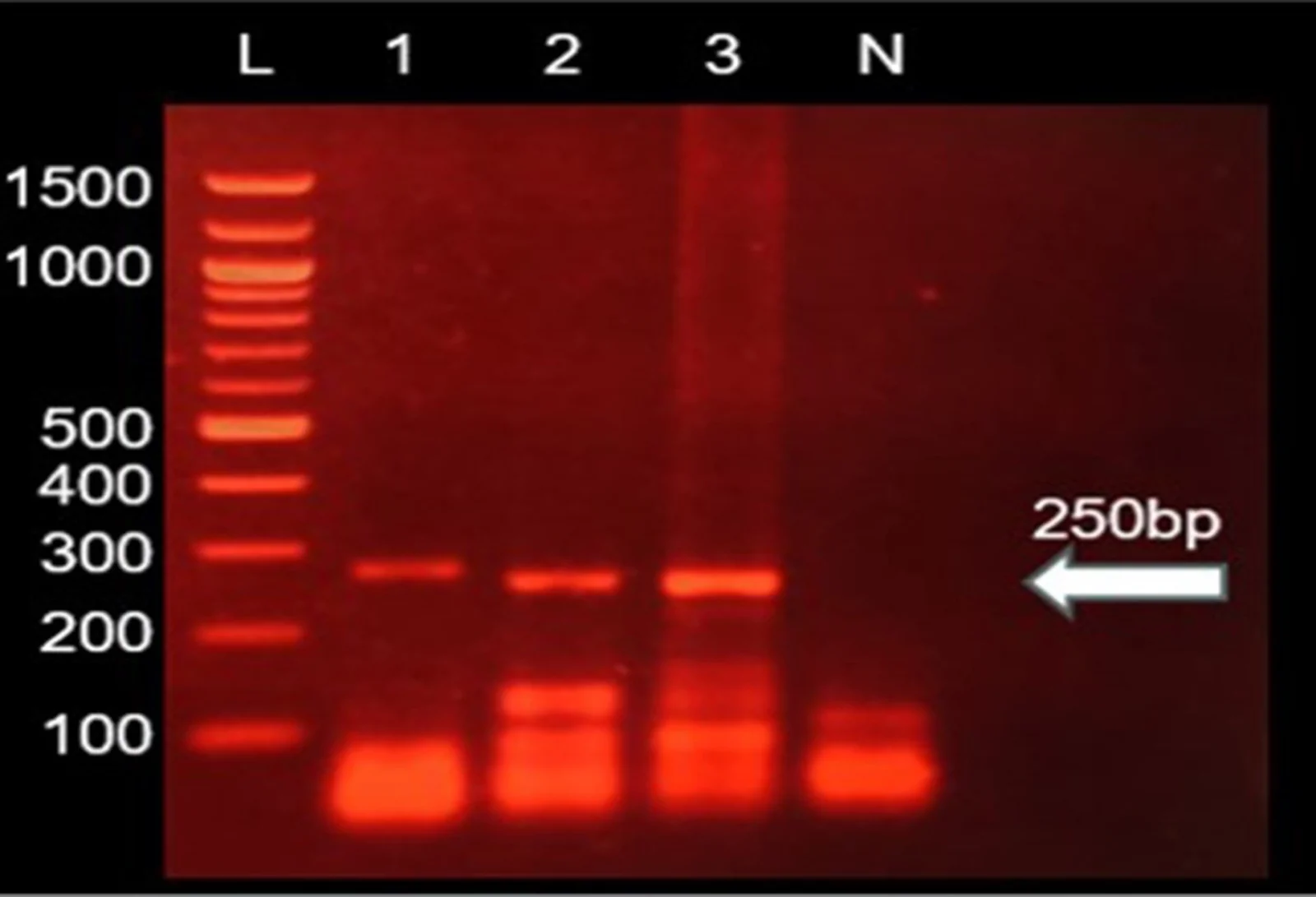
Amplification of the *S. enteritidis* genome of *Salmonella enteritidis* phage type 4 strain with a length of 250 bp PCR products. Lane L: DNA ladder (100 bp), lane 1: positive *S. enteritidis* stool sample, lane 2: positive *S. enteritidis* farm egg shell sample, lane 3: positive *L. monocytogenes* Baladi egg shell sample, and lane N: negative control.

As shown in Table 3; Fig. 1, L. *monocytogenes* DNA was detected in 44.82% and 58.62% of farm and Baladi eggshell samples, respectively, whereas its prevalence in egg contents was markedly lower, at 3.44% and 10.34%, respectively, with a statistically significant difference between shells and contents. Similarly, *S. enteritidis* DNA was identified in 34.48% of farm eggshell samples and 65.51% of Baladi eggshell samples, demonstrating a significant variation between the two groups (Fig. 2). Notably, *S. enteritidis* DNA was not detected in the contents of either farm or Baladi eggs.

**Table 3:** Prevalence of *L. monocytogenes* and *S. enteritidis* infections in egg types and parts.

Bacteria spp.	Egg types	Egg parts	No. of examined samples	Positive (%)	X_2_	*p* < 0.05
*L. monocytogenes*	Farm eggs	Shell	29	13(44.82%)	29.791	0.000^**^
		Content		1(3.44%)		
	Baladi eggs	Shell	29	17(58.62%)		
		Content		3(10.34%)		
*S. enteritidis*	Farm eggs	Shell	29	10(34.48%)	46.115	0.000^**^
		Content		0(0%)		
	Baladi eggs	Shell	29	19(65.51%)		
		Content		0(0%)		

(** highly significant).

#### Relation between L. monocytogenes infection in egg content and eggshell of the same egg

The data presented in Table 4 indicate that, among farm egg samples, 13 were positive for *L. monocytogenes* exclusively on the eggshells, while 15 samples were negative in both shells and contents. Only one sample showed contamination in the egg content despite a negative shell. Regarding Baladi egg samples, two samples were positive in both shells and contents. In addition, 15 samples were contaminated exclusively on the shells, whereas 11 samples were negative in both shells and contents. Similarly, only one sample exhibited contamination in the content with a negative shell.

**Table 4:** The relation between L. monocytogenes infection in the egg content and the egg shell of the same egg.

Egg type			Egg parts		Total	X_2_	p < 0.05
			Content				
			Positive	Negative			
Farm eggs	Shell	Positive	0	13	13	0.842	0.359
		Negative	1	15	16		
		Total	1	28	29		
Baladi eggs	Shell	Positive	2	15	17	0.089	0.765
		Negative	1	11	12		
		Total	3	26	29		

#### Correlation between the detected egg samples according to bacteria. Spp. infections

As shown in Table 5, among farm eggshell samples, 7 (24.13%) were positive for *L. monocytogenes* only, 4 (13.79%) for *S. enteritidis* only, and 6 (20.68%) for mixed infection. In contrast, farm egg content samples showed a markedly lower prevalence, with only 1 (3.44%) sample positive for *L. monocytogenes* and no detection of *S. enteritidis* or mixed infections. Similarly, in Baladi eggshell samples, 4 (13.79%) were positive for *L. monocytogenes* only, 6 (20.68%) for *S. enteritidis* only, and 13 (44.82%) for mixed infection. In egg contents, 3 (10.34%) samples were positive for *L. monocytogenes*, while no samples were positive for *S. enteritidis* or mixed infections. Overall, no statistically significant differences were observed between farm and Baladi eggs with respect to the detection of the investigated bacterial pathogens.

**Table 5:** Correlation between the detected egg samples according to bact. spp. infections.

Samples types	No. of examined samples	*Bact.* spp. positive samples (%)			*X*_*2*_	*p* < 0.05
		***L. monocytogenes only***	***S. enteritidis only***	**Mixed infection**		
Farm egg shell	29	7(24.13%)	4(13.79%)	6 (20.68%)	11.177	0.083
Farm egg content		1(3.44%)	0(0%)	0(0%)		
Baladi egg shell		4(13.79%)	6(20.68%)	13(44.82%)		
Baladi egg content		3(10.34%)	0(0%)	0(0%)		

### Molecular detection of L. monocytogenes and S. enteritidis in diarrheic patients

#### Prevalence of L. monocytogenes and S. enteritidis infections in diarrheic patients

In Table 6, the prevalence of *L. monocytogenes* and *S. enteritidis* infections were 11.76% and 41.17%, respectively, with a significant difference.

**Table 6:** Prevalence of *L. monocytogenes* and *S. enteritidis* infections in diarrheic patients.

No of examined samples	Bact. Spp.	Positive samples (%)	X_2_	*p* < 0.05
34	*L. monocytogenes*	4 (11.76%)	7.556	0.006^**^
	*S. enteritidis*	14 (41.17%)		

(** highly significance).

#### Prevalence of L. monocytogenes and S. enteritidis infections in diarrheic patients according to sex

As shown in Table 7, the prevalence of *L. monocytogenes* was higher in males (15%) than in females (7.14%), whereas *S. enteritidis* infections were more frequently detected in females (50%) compared to males (35%). However, these differences were not statistically significant between the two sexes for either pathogen.

**Table 7:** Prevalence of *L. monocytogenes* and *S. enteritidis infections* in diarrheic patient and associated risk factors.

Risk factors	Bacteria Spp.	No. of the examined samples		Prevalence No. (%)	X_2_	*p* < 0.05
Sex	L. monocytogenes	Male	20	3 (15%)	0.490	0.484
		Female	14	1 (7.14%)		
	S. enteritidis	Male	20	7 (35%)	0.765	0.382
		Female	14	7 (50%)		
Age	L. monocytogenes	5 m − 1Y	5	1 (20%)	0.437	0.933
		> 1Y − 5Y	10	1 (10%)		
		> 5Y − 10Y	11	1 (9.09%)		
		> 10Y − 16Y	8	1 (12.5%)		
	S. enteritidis	5 m − 1Y	5	1 (20%)	2.510	0.473
		> 1Y − 5Y	10	3 (30%)		
		> 5Y − 10Y	11	6 (54.54%)		
		> 10Y − 16Y	8	4 (50%)		
Symptoms	L. monocytogenes	With accompanying symptoms	21	2 (9.52%)	0.266	0.606
		Without accompanying symptoms	13	2 (15.38%)		
	S. enteritidis	With accompanying symptoms	21	7 (28.57%)	1.395	0.238
		Without accompanying symptoms	13	7 (53.84%)		

#### Prevalence of L. monocytogenes and S. enteritidis infections in diarrheic patients according to age

With respect to age, the highest prevalence of *L. monocytogenes* was observed in the 5 months–1 year age group (20%), followed by > 10–16 years (12.5%) and > 1–5 years (10%), while the lowest prevalence was recorded in the > 5–10 years group (9.09%). In contrast, *S. enteritidis* showed the highest prevalence in the > 5–10 years age group (54.54%), followed by > 10–16 years (50%) and > 1–5 years (30%), whereas the lowest prevalence was detected in the 5 months–1 year group (20%). However, no statistically significant differences were observed among the different age groups for either pathogen (Table 7).

#### Prevalence of L. monocytogenes and S. enteritidis infections in diarrheic patients according to symptoms

As shown in Table 7, L. *monocytogenes* and *S. enteritidis* were detected in 9.52% and 28.57% of diarrheic patients presenting with accompanying symptoms, respectively. In contrast, higher detection rates were observed among patients without accompanying symptoms, at 15.38% for *L. monocytogenes* and 53.84% for *S. enteritidis*. However, these differences were not statistically significant between the two groups.

According to Supplementary Table 1, among *L. monocytogenes*-infected patients, vomiting and abdominal pain were each reported in 20% of cases, whereas dehydration and fever were not observed (0%), with no significant associations identified. In comparison, among *S. enteritidis*-infected patients, vomiting and fever were each reported in 40% of cases, while dehydration and abdominal pain were more frequently observed, at 50% and 60%, respectively.

#### Demonstration of positive L. monocytogenes and S. enteritidis infections in diarrheic patients according to accompanying symptoms

Based on the data presented in Supplementary Table 2, 50% of *L. monocytogenes*-positive diarrheic patients exhibited both vomiting and abdominal pain, while fever and dehydration were not reported (0%). In contrast, 28.6% of *S. enteritidis*-positive patients presented with various combinations of symptoms, including vomiting, fever, dehydration, and abdominal pain. These manifestations occurred in different combinations across patients, such as vomiting with fever and dehydration, fever with abdominal pain, vomiting with dehydration and abdominal pain, and vomiting with abdominal pain, as well as isolated abdominal pain or dehydration. Notably, abdominal pain was the most frequently reported symptom, occurring in 85.7% of *S. enteritidis*-positive patients with accompanying symptoms.

## Discussion

*Listeria monocytogenes* is a significant foodborne pathogen responsible for listeriosis in humans^4^, largely due to its ability to persist in raw and ready-to-eat (RTE) foods stored under refrigerated conditions. For several decades, the contamination of eggs with *Salmonella* has posed a major challenge to public health authorities and the poultry industry worldwide. In Europe, eggs and egg products are among the most common vehicles of human salmonellosis, predominantly caused by *Salmonella enterica* serovar *Enteritidis*^19^.

Our findings demonstrated that *L. monocytogenes* DNA was detected in 55.2% of the examined egg samples, a prevalence higher than that reported by^20^(6.7%)^21^, (18.3%), and^14^(7%). Similarly, *S. enteritidis* DNA was identified in 50% of egg samples in the present study (Table 2). This prevalence exceeds earlier reports, including an overall incidence of 3.41%^22^, and 0.5% (27/5,548 pooled samples representing 33,288 eggs) documented in studies conducted in China^23^, where *S. enteritidis* was the predominant serotype. In contrast, another study has reported no detectable contamination with *Salmonella* spp. or *L. monocytogenes* in examined egg samples^1^. The comparatively higher prevalence observed in this study may be attributed to several factors, including geographical variation, differences in farm management and biosecurity practices, environmental and climatic conditions, sampling strategies, and the higher analytical sensitivity of PCR-based detection methods compared with conventional culture techniques.

As shown in Table 3, L. *monocytogenes* DNA was detected in 44.82% and 58.62% of farm and Baladi eggshell samples, respectively. In contrast, its prevalence in content was markedly lower, at 3.44% and 10.34%, respectively. These findings differ significantly from a previous report^24^, which found *L. monocytogenes* in 13.3% of pooled egg samples, exclusively on eggshells, with no detection in egg contents. Similarly, *S. enteritidis* DNA was identified in 34.48% and 65.51% of farm and Baladi eggshell samples, respectively, showing a significant variation between the two egg types. Notably, *S. enteritidis* DNA was not detected in the egg contents of either farm or Baladi eggs.

Consistent with our findings, *Salmonella* species were not isolated from the contents of Baladi hen eggs in studies by^25^ and^26^. In contrast, a previous study^27^, reported the isolation of *S. enteritidis* from one Baladi eggshell sample and one farm egg content sample, while another study^28^ detected *S. enteritidis* in 3.33% of both eggshell and content samples. Such variation may be attributed to differences in the health status of laying hens, the level of eggshell contamination and its subsequent penetration, as well as the possibility of transovarian *Salmonella* in eggs^29^.

As shown in Table 4, most *L. monocytogenes* detections in farm eggs were confined to the eggshell, with 13 samples testing positive on the shell only, while 15 samples were negative in both shell and content. Only one sample exhibited internal contamination despite a negative shell result. A similar trend was observed in Baladi eggs, where the majority of positive samples were limited to the shell (*n* = 15), and 11 samples were negative in both shell and content. However, two samples showed contamination in both shell and content, and one sample demonstrated internal contamination in the absence of shell positivity. These findings suggest that eggshell contamination does not necessarily translate into contamination of egg contents. This observation is consistent with previous studies conducted in Egypt, such as^20^ and^14^, which reported lower overall prevalence rates and similarly found that contamination was largely restricted to the eggshell, with minimal or no detection in egg contents.

The higher detection rates observed in the present study may be attributed to the use of direct PCR, a more sensitive technique compared to conventional methods. The predominance of shell-only contamination can be explained by the natural defense mechanisms of eggs. The eggshell, shell membranes, and antimicrobial components of egg albumen, such as lysozyme, ovotransferrin, and avidin, act as effective barriers that limit bacterial penetration and survival within the egg under normal conditions. Consequently, eggshell contamination alone should not be considered a reliable indicator of internal contamination^10,14,20,30,31^.

Nevertheless, the detection of *L. monocytogenes* in both shell and content in some samples suggests that bacterial penetration can occur. This may result from compromised shell integrity or unfavorable storage conditions, including high humidity, temperature fluctuations, prolonged storage, or exposure to high bacterial loads, all of which facilitate horizontal transmission from shell to content. Additionally, the occurrence of internal contamination in the absence of shell contamination indicates the possibility of vertical (transovarian) transmission, whereby the pathogen infects the reproductive tract of laying hens and contaminates the egg prior to shell formation^10,31^. Although less common, this route has been documented and provides a plausible explanation for such findings.

In Table 5, contamination of farm eggs was predominantly detected on the eggshell. Specifically, 7 (24.13%) samples were positive for *L. monocytogenes* only, 4 (13.79%) for *S. enteritidis* only, and 6 (20.68%) showed mixed infection. In contrast, egg contents showed minimal contamination, with only one sample (3.44%) positive for *L. monocytogenes* and no detection of *S. enteritidis* or mixed infection. A similar pattern was observed in Baladi eggs, where eggshell contamination was more frequent than internal contamination. Among eggshell samples, 4 (13.79%) were positive for *L. monocytogenes* only, 6 (20.68%) for *S. enteritidis* only, and 13 (44.82%) showed mixed infection. In comparison, egg contents revealed limited contamination, with 3 (10.34%) samples positive for *L. monocytogenes* and no detection of *S. enteritidis* or mixed infections. The present findings are in agreement with previous studies indicating that bacterial contamination of eggs is primarily confined to the shell, while internal contamination occurs less frequently due to the protective barriers of intact eggs. A previous study reported that contamination is largely localized on the eggshell surface, with limited penetration into the egg contents^31^. Similarly, another study demonstrated that eggshells commonly harbor foodborne pathogens, including *Salmonella*, whereas contamination of egg contents remains relatively rare, largely due to the antimicrobial properties of egg albumen and the integrity of shell membranes^30^.

The higher prevalence of mixed bacterial contamination observed in Baladi eggshells in the present study is consistent with^14^, who attributed increased microbial contamination in traditionally marketed eggs to inadequate sanitation, greater environmental exposure, and poor handling practices. In addition, the detection of *L. monocytogenes* alone in egg contents, without concurrent *S. enteritidis*, aligns with the findings of^20^, who reported that *L. monocytogenes* may occasionally be detected internally, while mixed infections are uncommon. This pattern suggests that internal contamination may arise from transovarian transmission or, less frequently, from bacterial penetration through the shell under unfavorable environmental conditions. Furthermore, a previous study emphasized that although external contamination of eggs is relatively common, internal contamination by foodborne pathogens remains infrequent due to the combined effect of physical and biochemical defense mechanisms inherent to eggs^10^.

In Table 6, the prevalence of *L. monocytogenes* and *S. enteritidis* infections among diarrheic patients was 11.76% and 41.17%, respectively, with a significant difference between them. The detected prevalence of *L. monocytogenes* (11.76%) was higher than that reported by^32^, who identified 9 (5.2%) positive samples using PCR. It was also higher than the prevalence reported in other countries, including 0% in Korea and China and 0.2% in Canada^33–35^. Similarly, the prevalence of *S. enteritidis* infections (41.17%) in the present study was higher than the rates reported by^36^ and^37^.

According to Table 7, the prevalence of *L. monocytogenes* infection was higher in males (15%) than in females (7.14%), although the difference was not statistically significant. This finding is consistent with^32^, who reported that males accounted for 5 cases (55.5%) of *L. monocytogenes* infections, while females represented 4 cases (44.4%). In contrast, another study from Sweden^38^ showed a slightly higher prevalence among females (52%) compared to males (50%). In the present study, *S. enteritidis*infections were more prevalent in females (50%) than in males (35%), with no statistically significant difference between the two groups. Conversely^37^, reported a nearly equal distribution, with 13 (48.1%) female and 14 (51.9%) male patients among the examined individuals.

As shown in Table 7, the highest prevalence of *L. monocytogenes* was observed in the 5 months − 1 year age group (20%), followed by > 10–16 years (12.5%) and > 1–5 years (10%), while the lowest prevalence was recorded in the > 5–10 years group (9.09%). However, no statistically significant differences were detected among the age groups. This finding is in partial agreement with^32^, who reported that 7 cases (77.7%) occurred in children younger than five years.

On the other hand, the present study showed that the highest prevalence of *S. enteritidis* was detected in the > 5–10 years age group (54.54%), followed by > 10–16 years (50%) and > 1–5 years (30%), whereas the lowest prevalence was observed in the 5 months–1 year group (20%). Similarly, no statistically significant differences were found among the age groups. These results are consistent with^36^, which reported that the 5–9 years age group had the highest detection rate (15.1%), followed by < 5 years (10.1%), while lower prevalence rates were recorded in older age groups, including 30–39 years (4.9%) and > 60 years (2.8%). The higher infection rates among children may be attributed to behavioral factors, such as close contact with asymptomatic carriers, frequent exposure to contaminated surfaces, and consumption of contaminated food or water^39,40^.

In Table 7, 9.52% and 28.57% of diarrheic patients with accompanying symptoms were infected with *L. monocytogenes* and *S. enteritidis*, respectively. In contrast, higher prevalence rates were observed among patients without accompanying symptoms, reaching 15.38% for *L. monocytogenes* and 53.84% for *S. enteritidis*, with no statistically significant differences between the two groups. According to Supplementary Table 1, a correlation analysis showed that vomiting and abdominal pain were each present in 20% of *L. monocytogenes*-infected cases. In contrast, dehydration and fever were not detected (0%) among infected patients. These findings differ from those reported by^32^, where *L. monocytogenes*-infected children commonly exhibited fever (88.8%), mucus in stool (77.7%), abdominal pain (44.4%), and blood in stool (11.1%). Similarly, studies have reported that *L. monocytogenes* gastroenteritis is commonly characterized by fever, diarrhea, headache, and other gastrointestinal symptoms such as nausea, vomiting, and abdominal pain, with a large proportion of patients experiencing at least one digestive manifestation^41,42^. Importantly, the relatively small number of *L. monocytogenes*-positive cases in the present study limits the strength of conclusions regarding clinical manifestations; therefore, these observations should be considered preliminary.

The observed clinical pattern suggests that *S. enteritidis* infection in diarrheic patients is commonly associated with gastrointestinal and systemic manifestations. Abdominal pain (60%) and dehydration (50%) appear to be the most prominent features, indicating significant intestinal involvement and fluid loss. Meanwhile, the moderate occurrence of vomiting and fever (40% each) reflects a typical inflammatory response to infection. Overall, these findings indicate that *S. enteritidis* may present with a combination of enteric symptoms and systemic signs, with abdominal pain being the most consistent clinical indicator in the studied patients.

## Study limitations

This study has several limitations that should be acknowledged. First, the detection of *L. monocytogenes* and *S. enteritidis* relied exclusively on PCR, without subsequent culture-based isolation and confirmation. Consequently, bacterial viability could not be assessed. Future studies should integrate molecular techniques with conventional culture methods to confirm the presence of viable organisms and enhance the epidemiological relevance of the findings. Second, further characterization of the detected bacteria was not performed. Analyses such as serotyping, detection of virulence-associated genes, antimicrobial susceptibility testing, and whole-genome sequencing were beyond the scope and available resources of the present study. Accordingly, future research incorporating bacterial isolation alongside comprehensive phenotypic and molecular characterization would provide a more robust understanding of the public health significance of these pathogens.

## Conclusion

The present study revealed a high molecular prevalence of *L. monocytogenes* and *Salmonella enterica* serovar *Enteritidis* in table eggs and stool samples from diarrheic patients in Assiut City, Egypt, suggesting a potential epidemiological link between contaminated eggs and human enteric infections. The higher detection rate of *L. monocytogenes* on eggshells compared with egg contents indicates that external contamination may represent a major route of bacterial transmission. Variations in pathogen prevalence according to age and sex were also observed among diarrheic patients, reflecting possible differences in susceptibility and exposure patterns. However, it is important to note that the detection of pathogen DNA indicates a potential public health concern rather than confirming the presence of viable bacteria or direct zoonotic transmission. Accordingly, strengthening biosecurity measures in laying hen farms, implementing routine microbiological surveillance, and promoting proper egg handling, storage, and cooking practices are essential to mitigate the risk of foodborne transmission. Furthermore, large-scale epidemiological and molecular studies are recommended to elucidate transmission dynamics and assess the genetic relatedness between poultry- and human-derived isolates, thereby supporting the development of effective prevention and control strategies.

## Supplementary Information

Below is the link to the electronic supplementary material.

Supplementary Material 1

## Data Availability

Datasets generated and/or analyzed during the current study are available from the corresponding author upon reasonable request.

## References

[1] Ghasemian Safaei, H., Hosseini, J. M., Sharifzadeh, A. & A, N. T. & The prevalence of bacterial contamination of table eggs from retails markets by Salmonella spp., Listeria monocytogenes, Campylobacter jejuni and Escherichia coli in Shahrekord, Iran. *Jundishapur J. Microbiol.* **4**, 249–253 (2011).

[2] Filiousis, G., Anders, J., Joachim, F. & Vincent, P. Food control short communication: prevalence, genetic diversity and antimicrobial susceptibility of Listeria monocytogenes isolated from open-air food markets in Greece. *Food Control*. **21**, 708–713 (2009).

[3] Pesavento, G., Ducci, B., Nieri, D., Comodo, N. & Nostro, A. L. Prevalence and antibiotic susceptibility of *Listeria* spp. isolated from raw meat and retail foods. *Food Control* **21**, 708–713 (2010).

[4] Shi, W., Qingping, W., Jumei, Z., Moutong, C. & Zéan, Y. Prevalence, antibiotic resistance and genetic diversity of Listeria monocytogenes isolated from retail ready-to-eat foods in China. *Food Control*. **47**, 340–347 (2015).

[5] Zhou, X. & Jiao, X. Investigation of Listeria monocytogenes contamination pattern in local Chinese food market and the tracing of two clinical isolates by RAPD analysis. *Food Microbiol.* **21**, 695–702 (2004).

[6] Koopmans, M. M., Brouwer, M. C., Vázquez-Boland, J. A. & van de Beek, D. Human listeriosis. *Clin. Microbiol. Rev.* **36**, e00060-00019 (2023). 36475874 10.1128/cmr.00060-19 PMC10035648

[7] Allerberger, F. Listeria: growth, phenotypic differentiation and molecular microbiology. *FEMS Immunol. Med. Microbiol.* **35**, 183–189 (2003). 12648835 10.1016/S0928-8244(02)00447-9

[8] Yücel, N., Cıtak, S. & Önder, M. Prevalence and antibiotic resistance of *Listeria* species in meat products in Ankara, Turkey. *Food Microbiol.* **22**, 241–245 (2005).

[9] Jeyaletchumi, P. et al. Detection of Listeria monocytogenes in foods. *Int. Food Res. J.* **17**, 1–11 (2010).

[10] Gantois, I. et al. Mechanisms of egg contamination by Salmonella Enteritidis. *FEMS Microbiol. Rev.* **33**, 718–738 (2009). 19207743 10.1111/j.1574-6976.2008.00161.x

[11] Patrick, M. E. et al. *Salmonella enteritidis* infections, United States, 1985–1999. *Emerg. Infect. Dis.* 7. 10.3201/eid1001.020572 (2004). 10.3201/eid1001.020572 PMC3322758 15078589

[12] EFSA. The community summary report on trends and sources of zoonoses, zoonotic agents, antimicrobial resistance and foodborne outbreaks in the European Union in 2006. *EFSA J.* **5**, 130r (2007).

[13] Braden, C. R. Salmonella enterica serotype Enteritidis and eggs: a national epidemic in the United States. *Clin. Infect. Dis.* **43**, 512–517 (2006). 16838242 10.1086/505973

[14] Sayed, M., Abdel-Azeem, M., Farghaly, M. & Hassanein, R. Using of PCR assay for identification of Listeria monocytogenes recovered from table eggs. *Vet. World*. **2**, 453–455 (2009).

[15] Jamali, H., Chai, L. C. & Thong, K. L. Detection and isolation of Listeria spp. and Listeria monocytogenes in ready-to-eat foods with various selective culture media. *Food Control*. **32**, 19–24 (2013).

[16] Soni, D. K., Singh, R. K., Singh, D. V. & Dubey, S. K. Characterization of *Listeria monocytogenes* isolated from Ganges water, human clinical and milk samples at Varanasi, India. *Infect. Genet. Evol.* **14**, 83–91 (2013). 23201044 10.1016/j.meegid.2012.09.019

[17] Pande, V. V., Devon, R. L., Sharma, P., McWhorter, A. R. & Chousalkar, K. K. Study of *Salmonella* Typhimurium infection in laying hens. *Front. Microbiol.* **7**, 203 (2016). 26941727 10.3389/fmicb.2016.00203 PMC4766288

[18] Sambrook, J. *Molecular cloning: a laboratory manual* (1989).

[19] Chousalkar, K., Gast, R., Martelli, F. & Pande, V. Review of egg-related salmonellosis and reduction strategies in United States, Australia, United Kingdom and New Zealand. *Crit. Rev. Microbiol.* **44**, 290–303 (2018). 28903617 10.1080/1040841X.2017.1368998

[20] Amin, F. Incidence Of Some Food- borne Pathogens In Table Eggs In Assiut City, Egypt. *Int. J. Trend Res. Dev.* **4** (6), 75–78 (2017).

[21] Jones, D., Curtis, P., Anderson, K. & Jones, F. Microbial contamination in inoculated shell eggs: II. Effects of layer strain and egg storage. *Poult. Sci.* **83**, 95–100 (2004). 14761090 10.1093/ps/83.1.95

[22] Gast, R. K., Jones, D. R., Guraya, R., Anderson, K. E. & Karcher, D. M. Research note: Contamination of eggs by *Salmonella enteritidis* and *Salmonella typhimurium* in experimentally infected laying hens in indoor cage-free housing. *Poult. Sci.* **100**, 101438 (2021). 34525442 10.1016/j.psj.2021.101438 PMC8445890

[23] Li, Y. et al. Prevalence and antimicrobial susceptibility of Salmonella in the commercial eggs in China. *Int. J. Food Microbiol.* **325**, 108623 (2020). 32339770 10.1016/j.ijfoodmicro.2020.108623

[24] Shaker, M., Hassanien, A. & E. & PCR technique for detection of some virulence associated genes in Listeria monocytogenes isolated from table eggs and clinical human samples. *Assiut Veterinary Med. J.* **61**, 219–225 (2015).

[25] Msallam, A. K. Occurrence of Salmonella spp. in hens eggs and their environment in selected farms in gaza strip. *Unpublished Master Thesis, Faculty of Science, the Islamic University–Gaza, Palestine* (2008).

[26] Arif, E. Isolation and Identification of Salmonella species from the table eggs in Sulaimani province. *AL-Qadisiya J. Vet. Med. Sci.* **12**, 24–27 (2013).

[27] Kamaly, R. M., Ewida, R. M. & Sayed, M. Molecular detection of Salmonella isolated from eggs and egg-based products. *J. Appl. Mol. Biology*. **1**, 1–11 (2023).

[28] Ma Elsherif, W., Hassan, M., THE IMPACT OF TEMPERATURES TO REDUCE, A. & THE RISK OF SALMONELLA ARIZONA AND SALMONELLA ENTERITIDIS IN TABLE EGGS. *Assiut Veterinary Med. J.* **59**, 146–154 (2013).

[29] De Buck, J., Van Immerseel, F., Haesebrouck, F. & Ducatelle, R. Colonization of the chicken reproductive tract and egg contamination by *Salmonella*. *J. Appl. Microbiol.* **97**, 233–245 (2004). 15239689 10.1111/j.1365-2672.2004.02294.x

[30] Musgrove, M. T., Jones, D. R., Northcutt, J. K., Harrison, M. A. & Cox, N. A. Impact of commercial processing on the microbiology of shell eggs. *J. Food Prot.* **68**, 2367–2375 (2005). 16300075 10.4315/0362-028x-68.11.2367

[31] De Reu, K. et al. Bacterial contamination of table eggs and the influence of housing systems. *World’s Poult. Sci. J.* **64**, 5–19 (2008).

[32] Abbasi, E., Amouzandeh-Nobaveh, A. & Ghaznavi-Rad, E. Frequency of Listeria monocytogenes Isolated from diarrhea samples of pediatric patients at central Iran. *Rep. Biochem. Mol. Biology*. **8**, 172 (2019). PMC6844618 31832442

[33] Schlech, I. I. I. Does sporadic Listeria gastroenteritis exist? A 2-year population-based survey in Nova Scotia, Canada. *Clin. Infect. Dis.* **41**, 778–784 (2005). 16107973 10.1086/432724

[34] Cheun, H.-I. et al. Infection status of hospitalized diarrheal patients with gastrointestinal protozoa, bacteria, and viruses in the Republic of Korea. *Korean J. Parasitol.* **48**, 113 (2010). 20585526 10.3347/kjp.2010.48.2.113 PMC2892565

[35] Shen, H. et al. The 12 gastrointestinal pathogens spectrum of acute infectious diarrhea in a sentinel hospital, Shenzhen, China. *Front. Microbiol.* **7**, 1926 (2016). 27965649 10.3389/fmicb.2016.01926 PMC5127809

[36] Shen, H. et al. Prevalence, serotypes, and antimicrobial resistance of Salmonella isolates from patients with diarrhea in Shenzhen, China. *BMC Microbiol.* **20**, 197 (2020). 32631309 10.1186/s12866-020-01886-5 PMC7339465

[37] Fardsanei, F. et al. Antimicrobial resistance patterns, virulence gene profiles, and genetic diversity of Salmonella enterica serotype Enteritidis isolated from patients with gastroenteritis in various Iranian cities. *Iran. J. Basic. Med. Sci.* **24**, 914 (2021). 34712421 10.22038/ijbms.2021.54019.12142 PMC8528249

[38] Carrique-Mas, J. et al. Febrile gastroenteritis after eating on-farm manufactured fresh cheese–an outbreak of listeriosis? *Epidemiol. Infect.* **130**, 79–86 (2003). 12613748 10.1017/s0950268802008014 PMC2869941

[39] Faulder, K. E., Simmonds, K. & Robinson, J. L. The epidemiology of childhood *Salmonella* infections in Alberta, Canada. *Foodborne Pathog. Dis.* **14**, 364–369 (2017). 28306338 10.1089/fpd.2016.2259

[40] Liang, B. et al. Prevalence, serotypes, and drug resistance of nontyphoidal Salmonella among paediatric patients in a tertiary hospital in Guangzhou, China, 2014–2016. *J. Infect. Public Health*. **12**, 252–257 (2019). 30466903 10.1016/j.jiph.2018.10.012

[41] Ooi, S. T. & Lorber, B. Gastroenteritis due to Listeria monocytogenes. *Clin. Infect. Dis.* **40**, 1327–1332 (2005). 15825036 10.1086/429324

[42] Dalton, C. B. et al. An outbreak of gastroenteritis and fever due to *Listeria monocytogenes* in milk. *N. Engl. J. Med.* **336**, 100–106 (1997). 8988887 10.1056/NEJM199701093360204

[43] Kawasaki, S. et al. Multiplex PCR for simultaneous detection of Salmonella spp., Listeria monocytogenes, and Escherichia coli O157: H7 in meat samples. *J. Food. Prot.* **68**, 551–556 (2005). 15771181 10.4315/0362-028x-68.3.551

[44] Ennaji, H., Timinouni, M., Ennaji, M. M., Hassar, M. & Cohen, N. Characterization and antibiotic susceptibility of *Listeria monocytogenes* isolated from poultry and red meat in Morocco. *Infect. Drug Resist.*10.2147/idr.s3632 (2008). 21694879 10.2147/idr.s3632 PMC3108722

[45] Soumet, C. et al. Evaluation of a multiplex PCR assay for simultaneous identification of Salmonella sp., Salmonella Enteritidis and Salmonella Typhimurium from environmental swabs of poultry houses. *Lett. Appl. Microbiol.* **28**, 113–117 (1999). 10063640 10.1046/j.1365-2672.1999.00488.x

[46] Krzyzanowski, F. et al. Quantification and characterization of Salmonella spp. isolates in sewage sludge with potential usage in agriculture. *BMC Microbiol.* **14**, 263 (2014). 25927729 10.1186/s12866-014-0263-x PMC4207900

