# Supplementary material for: Molecular investigation on Listeria monocytogenes and Salmonella enteritidis in table eggs and diarrheic patients
Source: Sci Rep. 2026 Aug 20;16:26177. doi: 10.1038/s41598-026-66486-1 (PMC13494040; doi:10.1038/s41598-026-66486-1)
Supplement: Supplementary file 1 [file 41598_2026_66486_MOESM1_ESM.docx]

**Supplementary Table 1. Prevalence of *L. monocytogenes* and *S. enteritidis* infections in diarrheic patients according to accompanying symptoms**

| **Accompanied**  **Symptoms** | **No. of examined samples** | ***L. monocytogenes*** | ***S. enteritidis*** |
| --- | --- | --- | --- |
|  |  | **Positive samples (%)** | **Positive samples (%)** |
| **Fever** | 5 | 0 (0%) | 2 (40%) |
| **Vomiting** | 5 | 1 (20%) | 2 (40%) |
| **Abdominal pain** | 10 | 1 (20%) | 6 (60%) |
| **Dehydration** | 4 | 0 (0%) | 2(50%) |

**Supplementary Table 2. Demonstration of positive *L. monocytogenes* and *S. enteritidis*** **infections in diarrheic patients according to accompanying symptoms**

| **Bacteria Spp.** | **Positive patients with accompanying symptoms** | **Accompanied symptoms** | | | |
| --- | --- | --- | --- | --- | --- |
|  |  | **Fever** | **Vomiting** | **Abdominal pain** | **Dehydration** |
| ***L. monocytogenes*** | 1 |  | √ |  |  |
|  | 1 |  |  | √ |  |
| **Total** | 2 | 0 (0%) | 1 (50%) | 1 (50%) | 0 (0%) |
| ***S. enteritidis*** | 1 |  |  | √ |  |
|  | 1 | √ |  | √ |  |
|  | 1 |  |  | √ |  |
|  | 1 | √ |  | √ |  |
|  | 1 |  | √ | √ | √ |
|  | 1 |  | √ | √ |  |
|  | 1 |  |  |  | √ |
| **Total** | 7 | 2 (28.6%) | 2 (28.6%) | 6 (85.7%) | 2 (28.6%) |
